# Multidimensional approach to formulating a specialized diet for northern corn rootworm larvae

**DOI:** 10.1038/s41598-019-39709-x

**Published:** 2019-03-06

**Authors:** Man P. Huynh, Bruce E. Hibbard, Stephen L. Lapointe, Randall P. Niedz, B. Wade French, Adriano E. Pereira, Deborah L. Finke, Kent S. Shelby, Thomas A. Coudron

**Affiliations:** 1 Division of Plant Sciences, University of Missouri, Columbia, Missouri 65211 USA; 2 Plant Genetics Research Unit, USDA-Agricultural Research Service, Columbia, Missouri 65211 USA; 3 U.S. Horticultural Research Laboratory, USDA-Agricultural Research Service, Fort Pierce, Florida 34945 USA; 4 North Central Agricultural Research Laboratory, USDA-Agricultural Research Service, Brookings, South Dakota 57006 USA; 5 Biological Control of Insects Research Laboratory, USDA-Agricultural Research Service, Columbia, Missouri 65203 USA

**Keywords:** Assay systems, Entomology

## Abstract

The northern corn rootworm (NCR), *Diabrotica barberi* Smith & Lawrence, is a major pest of maize (*Zea mays* L.). This pest has developed resistance to insecticides and adapted to crop rotation and may already be in the early stages of adaptation to toxins produced by *Bacillus thuringiensis* (Bt). Toxicity bioassays using artificial diet have proven to be valuable for monitoring resistance in many species, but no artificial diet has been developed specifically for NCR larvae. Toward this end, we first evaluated known *Diabrotica* diets to identify a starting media. We then developed a specialized diet for NCR using an iterative approach. Screening designs including 8 diet components were performed to identify the principal nutritional components contributing to multiple developmental parameters (survival, weight, and molting). We then applied mixture designs coupled with response surface modeling to optimize a blend of those components. Finally, we validated an improved NCR diet formulation that supports approximately 97% survival and molting, and a 150% increase in larval weight after 10 days of feeding compared with the best previously published artificial diet. This formulation appears suitable for use in diet bioassays as a tool for evaluating the resistance of NCR populations to insecticides.

## Introduction

The northern corn rootworm (NCR), *Diabrotica barberi* Smith & Lawrence, is a major insect pest of maize (*Zea mays* L.) in the North American Corn Belt. NCR and the western corn rootworm (WCR), *Diabrotica virgifera virgifera* LeConte, together, are responsible for more than $1 billion annually due to yield reduction and control costs in the United States^1^. Rootworm larvae feeding on corn roots cause yield loss from disruption to nutrient and water flow^2^ and can facilitate infection by soil pathogens^3,4^. Yield loss also occurs from unharvested grain due to the difficulty of mechanically harvesting lodged plants^5–7^. NCR is highly adaptive and has developed resistance and adaptation to several management tactics, including soil insecticides^8^ and crop rotation^9^. The latest management tools, transgenic maize hybrids expressing insecticidal proteins from *Bacillus thuringiensis* (Bt) Berliner, were recently documented to falter due to resistance of WCR^10–12^ to the toxins. Since the targeted pests of Bt maize are *Diabrotica* species including both WCR and NCR, the recent increase in NCR populations in some areas causes concern that a similar adaptation may occur with NCR to Bt maize and/or other management practices. In fact, NCR may already be in the early stages of adaptation to Bt^13^.

The United States Environmental Protection Agency (EPA) requires monitoring programs to detect the development of resistance in insect populations. Diet toxicity bioassays, whereby insects are exposed to toxins on artificial diet, can be a valuable component of such programs. However, this requires an artificial diet capable of supporting insect growth and development. To date, artificial diets of NCR for use in evaluating resistance to Bt maize in wild-type populations have been lacking. Because no artificial diet has been developed specifically for NCR, an artificial diet for southern corn rootworm (SCR), *Diabrotica undecimpunctata howardi* Barber, has been used in diet toxicity bioassays of a laboratory strain of NCR^14^. Development of an NCR diet to support development (e.g., survival and molting) would be helpful in detecting resistance development of NCR populations.

Diet development for *Diabrotica* species started with a formulation for larvae of SCR, which is a generalist that feeds on over 100 different plants. The first SCR formulation developed by Sutter *et al*.^15^ consisted of wheat germ and casein as key ingredients. This formulation supported SCR development from egg to adult, but development was slower and fecundity was lower for larvae reared on the diet compared with those fed corn roots^15^. SCR diet improvements were reported by Rose and McCabe^16^ and Marrone *et al*.^17^ by altering linseed oil, sucrose, antibiotics, and potassium hydroxide. The newest formulation improved SCR development and approached that of larvae reared on corn roots after 6 generations of selection for larval vigor and production of adults on the diet^17^. The SCR diet has been used in WCR diet bioassays, although growth rates for WCR were poorer than that of SCR and microbial contamination was a major issue^18^. Later, several modifications of this diet were made by industry and a high level of antibiotics was added into the SCR diet to avoid contamination^19^.

An initial formulation for WCR larvae was made by Pleau *et al*.^20^ with modifications made to a diet for SCR^17^ by adjusting amounts of several ingredients (i.e., wheat germ, linseed oil, and potassium hydroxide), removing formalin and adding corn root powder. The Pleau *et al*. diet doubled the weight of WCR larvae compared with larvae reared on the SCR formulation^20^. Later, the Pleau *et al*. diet was improved by Huynh *et al*.^21^, which resulted in a formulation that further increased the weight of WCR larvae and increased both survival and molting after 11 days compared with the first WCR formulation. We utilized response surface modeling to identify and then optimize the key ingredients in the Pleau *et al*. diet to develop the improved diet^21^. The Pleau *et al*. diet was improved by altering several components (i.e., sucrose, wheat germ, casein, cellulose, corn root powder, linseed oil, and agar) and adding wheat germ oil. The Huynh *et al*. diet had nearly zero contamination after 11 days when clean laboratory practices were applied^21^, but contamination was also eliminated from 5 other WCR diets using the same techniques^22^. This diet was also compatible with all current Bt proteins targeting WCR larvae^23^.

Past approaches to formulate and develop insect diets involved empirical one-factor-at-a-time (OFAT) and multivariate experiment designs based on mixture designs. For OFAT designs, each diet component was varied independently. Because changing the amount of one factor is confounded with changes in the proportions of other ingredients in a mixture, the OFAT design is not capable of identifying an optimal blend of diet components^24^. The multivariate geometric approach in combination with a mixture design allows simultaneous varying of multiple diet components and reveals important interactive effects of diet components on several measured responses at a same time^21,25,26^. Henri Scheffé^27^ developed mathematical models specifically for response surface modeling based on mixture designs and the corresponding computations are simplified by modern software for design of experiments^25,28^. The application of response surface methodology combined with a mixture design has been of great value for diet improvement and optimization for WCR^21^ and another coleopteran insect^25,29^.

For the current paper, we first compared the performance of 5 published *Diabrotica* artificial diets for rearing NCR and identified key diet ingredients in the best diet in a 8-component experiment. We then screened 8 protein sources to identify best options. Finally, we optimized specific blends to obtain our desired responses. We combined the response surface method with a mixture design to identify the main drivers of larval development by maximizing larval performance (larval survival, development, and weight) while limiting diet contamination compared with the performance of NCR on existing diets developed for related species (WCR and SCR).

## Results

### Comparison of five rootworm diets

**Figure 1:**
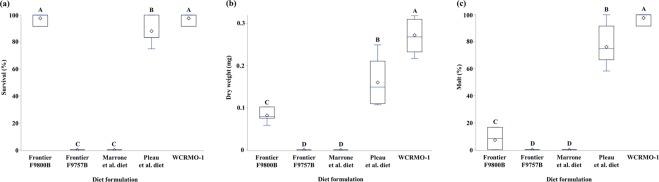
Percentage survival (**a**), average dry weight (**b**), percentage molting to 2^nd^ instar (**c**), of northern corn rootworm larvae fed different diet formulations for 10 days. Frontier SCR diets (F9800B & F9757B, Frontier Newark, DE), the Marrone *et al*. diet^17^, the Pleau *et al*. diet^20^, the WCRMO-1 diet^21^. Bars with different letters are significantly different (*P* < 0.0001).

Larval survivorship to 10 days was significantly different (*P* < 0.0001, *F*_4,24_ = 247.4) among the five tested diets (Fig. 1a). There was no significant difference between WCRMO-1 and Frontier 9800B. The Pleau *et al*. diet had significantly lower survivorship than WCRMO-1 and Frontier 9800B, whereas no larvae survived to 10 days when reared on Frontier 9757B or the Marrone *et al*. diet. Average larval dry weight after 10 days varied significantly across diets (*P* < 0.0001, *F*_4,24_ = 122.6). The WCRMO-1 diet had significantly higher larval dry weight than other diets, while the Frontier F9800B diet had significantly lower larval dry weight than the Pleau *et al*. diet (Fig. 1b). Significantly more NCR larvae molted from 1^st^ to 2^nd^ instar when reared on WCRMO-1 than on any other diet (*P* < 0.0001, *F*_4,24_ = 189.5) (Fig. 1c). The Pleau *et al*. diet had more successful larval molts compared with Frontier F9800B. Overall, WCRMO-1 supported better NCR larval performance than other diets tested.

### 8-component screening experiment for basic nutrition profile

**Figure 2:**
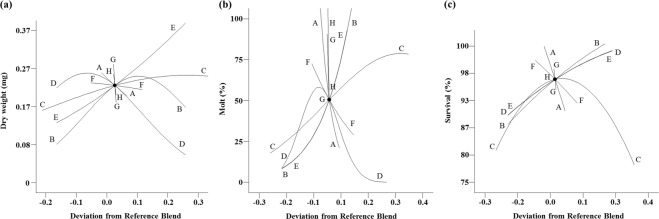
Cox plot of larval responses to deviations from a reference blend diet in the proportions of ingredients in an 8-component mixture experiment. (**a**) weight, (**b**) molting, (**c**) survival. Reference blend proportions: agar = 0.046, sucrose = 0.203, wheat germ = 0.253, casein = 0.203, cellulose = 0.203, corn root: 0.075, wheat germ oil = 0.009, linseed oil = 0.009. A: agar, B: sucrose, C: wheat germ, D: casein, E: cellulose, F: corn root powder, G: wheat germ oil, H: linseed oil. The slope of the line shows the inverted direction and magnitude of the influence of the ingredient on measured responses. The orientation of the graphs is inverted from what is normally expected, so effects that appear negative are positive.

The 8-component mixture design produced significant response surface models for all three measurement criteria; larval weight (*P* < 0.0001, *F*_9,13_ = 37.9), molting (*P* < 0.0001, *F*_8,14_ = 47.3), and survival (*P* < 0.0001, *F*_8,14_ = 19.1). Eight diet components were varied simultaneously: agar, sucrose, wheat germ, casein, cellulose, corn root powder, wheat germ oil and linseed oil. Models for weight and survival had insignificant lack of fit whereas there was a significant lack of fit for molting due to a very small value of pure error. All models were improved by stepwise regression. Values for R^2^, R^2^_adj_ and R^2^_pred_ varied by less than 0.2 (see Supplementary Table S5), indicating good predicted models. The relationships between diet ingredients and larval performance (Fig. 2), depict the effect of increasing the proportion of one component in relation to a reference blend while the relative proportions of all of the other diet components are kept constant^30,31^ and are called Cox plots. The Cox plot was generated by inverted high and low values, which showed that the slope of the line indicated the inverted direction and magnitude of the influence of the individual factors on the measured response variable, e.g., larval weight. Models for weight and molting indicated that casein, corn root powder and two lipid components had the greatest effects on improving these criteria (Fig. 2a,b) whereas a model for survival revealed that wheat germ, corn root powder and wheat germ oil were the primary drivers (Fig. 2c). Increases in the two lipid components resulted in minor improvements to WCR larval performance^21^. Casein was the main protein source of the diet formulation, and was an important driver for weight and molting. Based on that observation, we proceeded to test alternative protein sources.

### 8-protein mixture experiment

**Figure 3:**
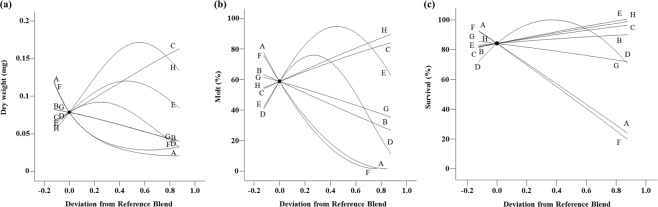
Cox plot of larval responses to deviations from a reference blend diet in the proportions of ingredients in an 8-protein screening experiment. (**a**) weight, (**b**) molting, (**c**) survival. Reference blend proportions: corn gluten meal = cottonseed meal = casein = plant protein = whey protein = perfect amino = yeast extract = egg powder = 0.375. A: corn gluten meal, B: cottonseed meal, C: casein, D: plant protein, E: whey protein, F: perfect amino, G: yeast extract, H: egg powder. The slope of the line indicates the direction and magnitude of the influence of the ingredient on measured responses.

The protein screening design yielded significant response surface models for larval weight (*P* < 0.0001, *F*_12,17_ = 35.6), molting (*P* < 0.0001, *F*_9,20_ = 16.4), and survival (*P* < 0.0001, *F*_8,21_ = 33.7) by varying 8 different protein sources: corn gluten meal, cottonseed meal, casein, plant protein, whey protein, perfect amino, yeast extract, and egg powder (see Supplementary Table S6). Models for weight had insignificant lack of fit whereas models for molting and survival had significant lack of fit due to very small values of pure error. R^2^, R^2^_adj_ and R^2^_pred_ of all models were clustered with differences <0.3. The relationships between protein sources and larval performance (Fig. 3) indicated animal protein sources (casein, egg powder, and whey protein) had the greatest effects on improving all three criteria of larval performance (Fig. 3a–c). In addition, plant protein had positive effects on larval performance and cottonseed meal had positive effect on survival. In contrast, corn gluten meal, perfect amino, and yeast extract had negative effects on all developmental traits. In Fig. 3, the slope shows the direction and magnitude of the impact of each ingredient on all measured responses.

### 3-protein mixture-amount experiment

**Figure 4:**
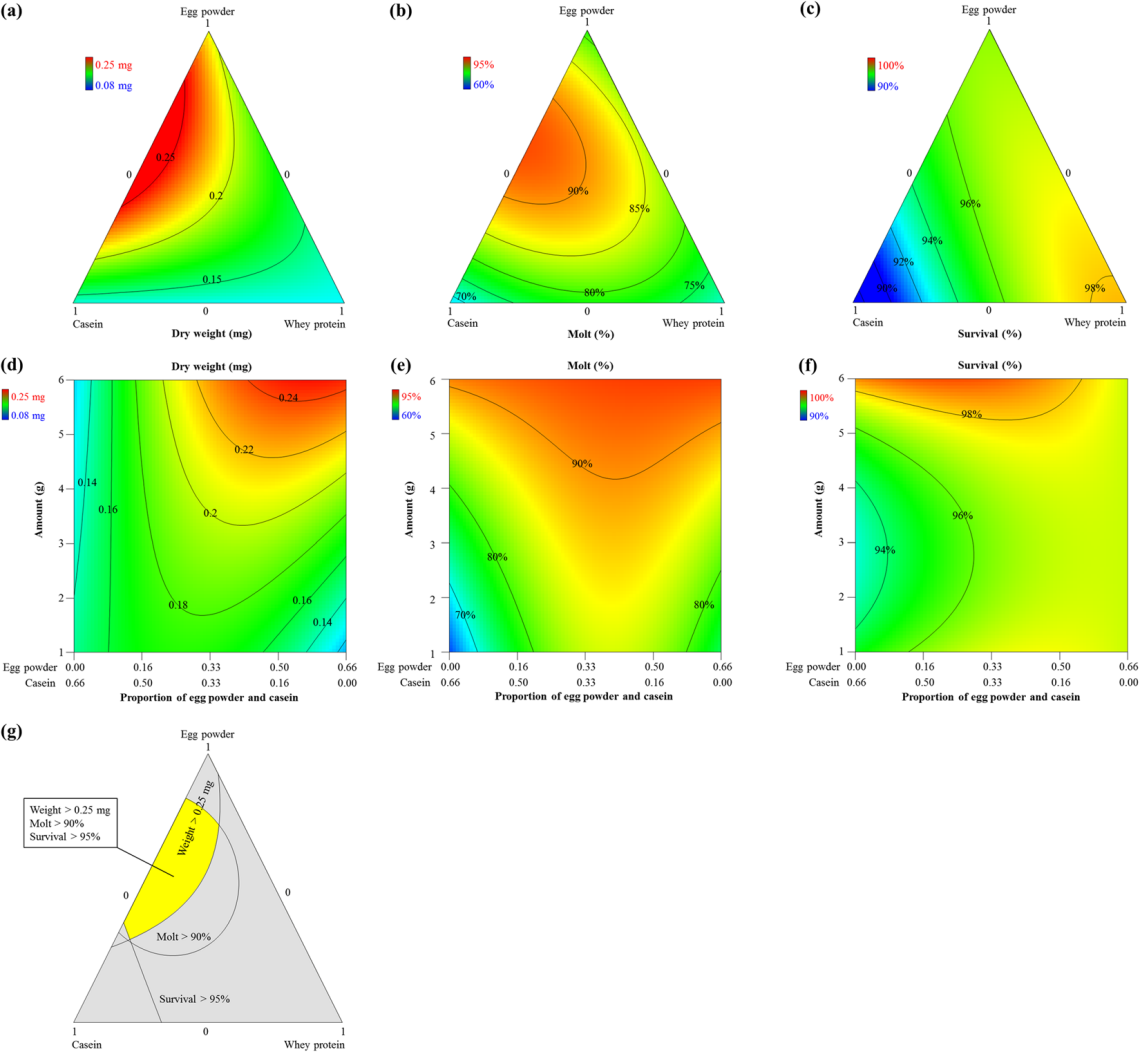
Contour plots of larval responses to a mixture-amount design of casein: egg powder: whey protein at 10 days post infestation. (**a**) weight, (**b**) survival, and (**c**) molting. (**d**–**f**) interaction of egg powder, casein and concentration on (**d**) weight, (**e**) survival, and (**f)** molting; proportions of whey protein = 0.34. Color bars display the magnitude of the measured response. (**g**) Overlaid optimization plot of larval responses; yellow area represents the optimum region for weight, molting, and survival that are obtained when three ingredients (i.e., egg powder, casein, whey protein) are used.

The 3-protein mixture-amount was used to identify an optimal 3-component blend for larval weight (*P* < 0.0001, *F*_5,25_ = 10.2), molting (*P* < 0.0001, *F*_8,22_ = 14.1), and survival (*P* < 0.0001, *F*_6,23_ = 8.8) using: casein, egg powder, and whey protein (see Supplementary Table S7). R^2^, R^2^_adj_ and R^2^_pred_ of all models were clustered with differences <0.3. Lack of fit of models for weight was significant due to very small values of pure error, while models for molting and survival had insignificant lack of fit. The relationships between protein sources and larval performance were shown in ternary plots (Fig. 4). Possible combinations of proportions of the mixtures of three components were revealed in the ternary plots. The magnitude of the response variables is coded in color and can be envisioned as perpendicular to the page as indicated by labelled isobars (Fig. 4a–c).

Models for weight and molting indicated that a blend of casein and egg powder was optimal for maximum weight and molting (Fig. 4a,b). High proportions of casein resulted in a decrease in all developmental traits. Whey protein had positive effect on survival (Fig. 4c), but a negative effect on weight and molting when added as a component of the protein mixture. Results also showed very strong blending effects of egg powder and casein on larval weight and molting, but an antagonistic blending effect of these components on survival (see Supplementary Fig. 1). Such a trade-off between survival and weight gain has been shown to occur in other beetles reared on artificial diets^32^.

The contour plots (Fig. 4d–f) represent the interactive effects of casein and egg powder and the amount of mixture while proportions of whey protein to diet mixture were constant at 0.34:1. The labelled isobars and color indicate the magnitude of the measured response. All contour plots indicated that a higher total amount of the mixture could yield a better formulation. Ratios of egg powder: casein at 4:1, 1:1, 3:1 are predicted to produce the best formulation for weight, molting and survival, respectively (Fig. 4d–f).

### Contamination

Since all experiments had minor contamination (<1%), there was no evidence for a relationship between contamination and diet ingredients. Similarly low contamination rates were seen in Huynh *et al*.^21^, Ludwick *et al*.^23^, and Meihls *et al*.^22^.

### Diet optimization

The diet development for NCR larvae produced a formulation, referred to hereafter as NCRMO-1 (Table 1). This formulation was calculated with Design-Expert^TM^ by optimizing key ingredients based on the integrated evaluation of all developmental traits for WCR. An overlay graph comprised of the contour plots from each response laid on top of each other was generated to identify a region where all measured responses were simultaneously maximized (Fig. 4g).

**Table 1:** An artificial diet for NCR larvae.

Ingredients	WCRMO-1^21^	NCRMO-1
Egg powder	−	+
Glucose	−	+
Casein	+	+
Wheat germ (raw, ground)	+	+
Cellulose	+	+
Agar	+	+
Corn root powder	+	−
Sucrose	+	−
Linseed oil, raw	+	−
Wheat germ oil	+	−
Cholesterol	+	+
Wesson’s salt mix	+	+
Vanderzant Vitamin mix	+	+
Methyl paraben	+	+
Sorbic acid	+	+
Potassium hydroxide (10%)	+	+
Streptomycin (12.8 mg/ml)	+	+
Chlortetracycline (10.0 mg/ml)	+	+
Distilled water	+	+
Green food coloring	+	+

### Model validation and diet improvement

**Figure 5:**
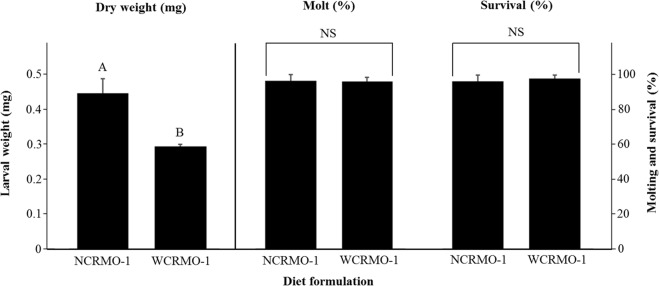
Larval dry weight, percent successful completion of molt, and survival for northern corn rootworm larvae reared on NCRMO-1 and WCRMO-1 diets for 10 days. Means within bars followed by different letters are significantly different (*P* < 0.05). Mean ± SEM.

The NCRMO-1 diet yielded better larval performance compared with the best previously published diet for WCR, WCRMO-1. The formulation for NCR larvae was modified from WCRMO-1, with several modifications including the elimination of corn root powder, linseed oil and wheat germ oil, substitution of sucrose with glucose, and addition of egg powder (Table 1). Other ingredients were at the same level as in WCRMO-1^21^. After 10 days of feeding on NCRMO-1, larval dry weight, survival and molting rate were 0.44 mg, 97%, and 97% compared to 0.28 mg, 98% and 96%, respectively when reared on WCRMO-1 (Fig. 5).

## Discussion

**Figure 6:**
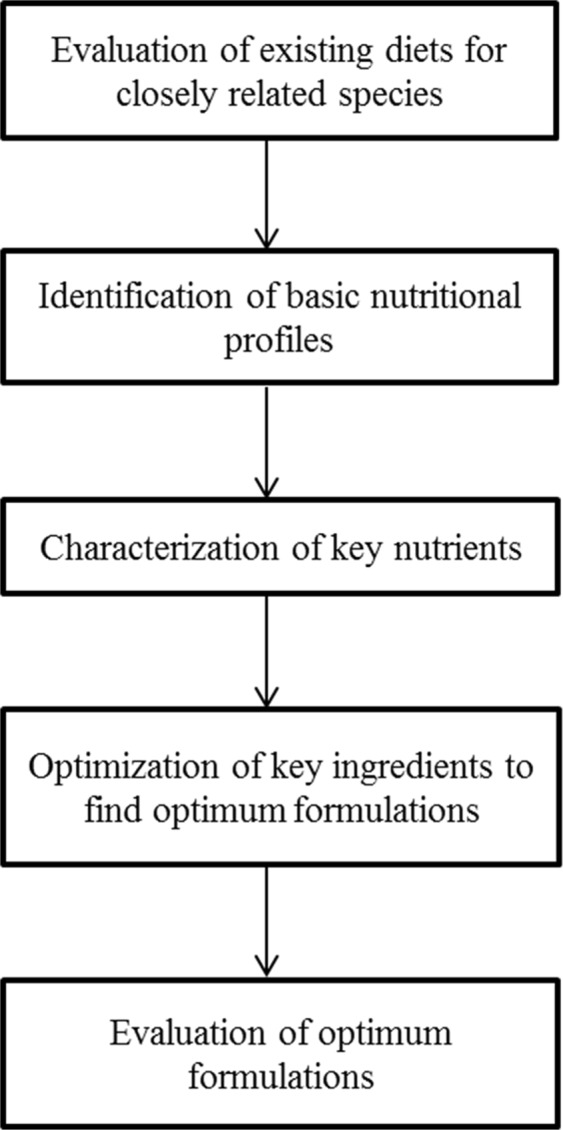
Flow chart for diet development algorithm.

We applied a multivariate geometric approach in combination with an *n*-dimensional mixture design to develop the first artificial diet formulated specifically for rearing NCR. The algorithm used consisted of five steps including evaluation of existing diets, identification of basic nutritional profiles, exploration of key nutrients, maximizing key ingredients, and evaluation of formulations, as presented in Fig. 6. The application of geometric and mathematical approaches including response surface modeling^33^, response surface modeling combined with *n*-dimensional mixture design^21,25,29^, and orthogonal experimental designs^34^ have been used for improvement of existing diets for other insect species. By applying the response surface methodology based on a multivariate mixture design, we identified key ingredients and then optimized the key ingredients to create the best formulation that maximized life history criteria (survival, molting, and weight) for NCR.

Our results indicated that protein ingredients were key components that had the greatest effect on larval performance, i.e., changing the concentration of these ingredients strongly affected the life history parameters. In contrast, other ingredients (wheat germ, cellulose, sucrose, linseed oil, wheat germ oil, and agar) had little or negative contributions on the overall response parameters (Fig. 2). We found that NCR larvae performed better with animal proteins compared with 8 other protein sources tested, including plant and yeast proteins. The addition of animal protein sources (casein, egg powder, and whey protein) and plant protein improved all measured responses (weight, molting, and survival) whereas the inclusion of other protein ingredients (corn gluten meal, cottonseed meal, perfect amino, and yeast extract) resulted in a decrease in all three measured responses, except for a positive effect of cottonseed meal on survival (Fig. 3a–c). The nonlinear blending effects indicated that a blend of casein and egg powder contributed the most to improved insect performance compared with all combinations of the three animal protein sources (Fig. 4d–f). Casein at high proportions (Fig. 4c) caused a decrease in survival when changed in combination with egg powder and whey protein. Our results revealed that wheat germ had a complex relationship with the life history parameters. Wheat germ at high proportions improved survival, but caused a decrease in molting (Fig. 2b,c). Similar effects of wheat germ on insect performance have been reported previously for WCR larvae^20,21^. Wheat germ is documented as a key ingredient for nutritional value, digestibility, bioavailability, and palatability, but at high proportions can cause detrimental effects on insect development^35^.

We found that corn root powder was a key ingredient that had a strong positive influence on life history parameters. The addition of corn root powder increased all larval performance parameters such as weight, molting and survival (Fig. 2a–c). The important role of corn root powder was also documented in WCR diets, whereas an increase in the proportion of root powder improved molting and survival but also caused a decrease in weight when added at high concentrations^20,21^. Corn roots contain host recognition cues and feeding stimulants, but also contain factors which have a repellent effect on WCR larvae^36–38^. Corn root powder is not currently available for purchase, making it a limiting factor for users. In this study, we identified, characterized and optimized key protein sources (Figs 3 and 4) that allowed us to eliminate corn root powder, making our formulation more widely applicable.

Our results revealed significant differences in NCR larval development among rootworm diets (Figs 1 and 5). WCRMO-1, a superior diet for WCR^21^, supported NCR larval performance better than SCR diets and another WCR diet. This is possibly because both WCR and NCR are nearly monophagous on corn roots, although they can also survive on some grass species^39,40^. There was zero NCR survival to day 10 when feeding on diets containing approximately 1% formalin, e.g., Frontier F9757B and Marrone *et al*. diets. A detrimental effect of formalin on weight was reported previously for WCR. Larval weight reduced to half when feeding on diets with an addition of 1% formalin^20^. In contrast, at 10 days post-infestation, survival and molting rate of NCR larvae reared on NCRMO-1 (without formalin) were approximately 97%. Larval dry weight increased 150% with NCRMO-1 compared with WCRMO-1 (Fig. 5).

Although the ultimate goal of insect artificial diet research might be to serve as a comparable substitute for natural foods^25^, only a few diet formulations can be considered to have achieved that goal over the past 60 years^35^. However, artificial diets are also desired to enable the study of insect species of interest^41–43^, to reduce labor or space^41^, to reduce time for host plant rearing^44^, and to provide a standardized diet for insect bioassays^21,23^. This is the first report of a diet formulation developed specifically for NCR larvae. NCRMO-1 supported high survival, weight, and percentage of larvae molted to second instar after 10 days. Additionally, this formulation had basically zero contamination. Consequently, this formulation is suitable for use in diet bioassays for monitoring resistance programs of NCR, and for the study of basic NCR biology and physiology.

## Materials and Methods

### Insects

NCR eggs (a diapausing strain) were obtained from the North Central Agricultural Research Laboratory in Brookings, South Dakota. Eggs were surface-treated using a procedure described by Pleau *et al*.^20^ (see Supplementary Methods).

### Diet preparation

Frontier SCR diets (F9800B & F9757B, Frontier, Newark, DE) were purchased and poured according to procedures by manufacturers. Other diets including the Marrone *et al*. diet, the Pleau *et al*. diet and the WCRMO-1 diet were made using a procedure described in Pleau *et al*.^20^, with some modifications by Huynh *et al*.^21^ (see Supplementary Methods).

### Insect diet bioassays

The diet bioassays were conducted as described in Huynh *et al*.^21^. All materials used in the diet assays were surface-treated via exposure to UV light for 10 min in a biological cabinet. Each formulation was tested in a 12-well row of a 96 well plate and values from the 12 sub-samples were averaged to yield a single replicate. Each formulation was replicated at least 4 times. Eight different formulations were randomly assigned on the plate. One larva (<24 h after hatching) was placed in each well of the diet plate using a fine paintbrush. A sealing film (TSS-RTQ-100, Excel Scientific, Inc., Victorville, CA) was used to cover the plate and one vent hole was made per well using an insect pin (size 0). The plates were stored at 25 °C in darkness for 10 days. Collections made at 10 days were used to determine differences in survival, weight and molting to the 2^nd^ instar. Larval survival, time of molt to 2^nd^ instar, and evidence of diet contamination were recorded daily. Live larvae were collected and pooled within a replication into 95% ethanol, dried in an oven (602752, Blue M Therm Dry Bacteriological Incubator) at 50 °C for 2 days, and then were weighed using a micro balance (MSU6.6S-000-DM, Sartorius Lab Instruments GmbH & Co. KG, Goettingen, Germany).

### Experimental approach

An iterative approach was used to develop an artificial diet for NCR. An initial experiment was conducted by comparing five current rootworm diets. A screening design was conducted to explore the nutritional profile of NCR by varying 8 components using a mixture design. Polynomial equations were generated to describe the impact of diet ingredients on the measured responses (i.e., larval weight, molting, survival, and contamination). A screening experiment was used to characterize the effects of 8 different protein sources on the measured responses using an 8-component mixture design. A mixture-amount design^45^ was also used to identify an optimum formulation of 3 protein ingredients that had the greatest effect on the measured responses. The predicted optimal blend was then validated in a comparison experiment.

### Comparison of five rootworm diets

Three SCR diets included the two Frontier diets (F9800B & F9757B, Frontier, Newark, DE) plus the Marrone *et al*. diet^17^. The two WCR diets included Pleau *et al*. diet^20^ and WCRMO-1^21^. An improved formulation was chosen based on overall larval performance (i.e., larval weight, molting, and survival) after 10 d of feeding in diet assays.

### 8-component mixture design for basic nutrition profile

Since the WCRMO-1 diet was the best of the five public rootworm diets evaluated, improvements started from this diet. It consisted of 18 diet ingredients^21^. An exploratory experiment to identify the basic nutritional profile of NCR simultaneously varied 8 diet components including casein, cellulose, corn root powder, wheat germ, wheat germ oil, linseed oil, and agar. Other ingredients (vitamin and salt blends, preservatives and antibiotics) were kept constant at the levels in the WCRMO-1^21^. No effort was made to de-convolute the mixtures of Vanderzant vitamin and Wesson salt that are widely used in insect diets. The exploratory design was created with Design-Expert (v.10.0, Stat-Ease, Inc., Minneapolis, MN) resulting in 24 design points including vertex, center, 7-blend, and axial check blend points (see Supplementary Table S1)^28^. The design included 7 model, 11 lack of fit, and 5 pure error degrees of freedom^46^.

### 8-protein screening design

The 8-component mixture experiment indicated casein, corn root powder, lipid ingredients and agar had positive effects on both NCR larval weight and molting. Survival was removed as a criterion due to more than 90% survival across almost all design points. Lipid ingredients (wheat germ oil and linseed oil) were removed due to their minor contributions in WCR diets^21^. Glucose was substituted for sucrose because glucose is a main component of sugar in corn roots^36^. In this study, 8 different protein sources were concurrently screened to characterize their contributions to larval performance (weight, molting, and survival) by constructing an I-optimal mixture design^47,48^. This design consisted of 32 design points with 7 model, 17 lack of fit and 5 pure error degrees of freedom (see Supplementary Table S2). Concentrations of other ingredients were the same as for WCRMO-1 (see Supplementary Table S3).

### 3-protein mixture design

The 3-proteins responsible for the largest effects on NCR larval weight, molting, and survival were used to construct a D-optimal mixture-amount design^45,49^ sufficient for a Scheffé quadratic-quadratic polynomial response surface model. This design consisted of 32 design points with 17 model, 6 lack of fit and 8 pure error degrees of freedom (see Supplementary Table S4). Other ingredients were kept constant as shown in see Supplementary Table S3. Optimal blends were predicted using overlay response surface plots combined with a simplex hill-climbing algorithm included in Design-Expert™ software^25^.

### Diet optimization

A formulation for maximizing larval performance (survival, molting, and weight) was calculated with Design-Expert^TM^ (Stat-Ease, Inc., Minneapolis, MN). This software used overlay response surface and direct search methods^50^ to maximize the desirability function^51^.

### Model validation

All three measures of larval performance (survival, molting, and weight) identified by the response surface mixture model were compared with those of larvae reared on WCRMO-1 diet^21^.

### Statistical analyses

Survival and molting data were generated by dividing the number of live larvae and successful larval molts from 1^st^ to 2^nd^ instar per replicate, respectively, by the initial number of larvae infested and multiplying by 100 to obtain percentages. Weight data were generated by dividing total dry weight per replicate by the number of live larvae while dead larvae were recorded as 0 in weight.

In the diet comparison experiments, measured parameters of larval performance on the rootworm diets at 10 days after infestation were analyzed as a randomized complete block design using PROC MIXED in SAS^52^. All percent variables were arcsine square-root transformed prior to the analysis to meet assumptions of normality and homoscedasticity.

In the mixture experiments, all possible models from 1^st^ degree to 4^th^ degree polynomials for each measured response of larval performance (larval survival, proportion of successful larval molts and larval weight) were generated with Design Expert^®^_._ Model selection as described in Lapointe *et al*.^25^. Briefly, the criteria included a lack of aliased terms, low residual values, low model *P*-value, nonsignificant lack of fit, low standard deviation, high R^2^, R^2^_adj_ and R^2^_pred_ ^25^, close agreement between R^2^_adj_ and R^2^_pred_, and a low PRESS value^46,53^. When two or more models were satisfactory, the selected model was then further evaluated according to adequacy tests as described by Anderson and Whitcomb^28,54^.

## Supplementary information

Supplementary Information

## Data Availability

All pertinent data are found in the figures and tables. Requests for data and additional information should be submitted to the corresponding author.
