# Author Correction: Multidimensional approach to formulating a specialized diet for northern corn rootworm larvae

**DOI:** 10.1038/s41598-020-57440-w

**Published:** 2020-01-15

**Authors:** Man P. Huynh, Bruce E. Hibbard, Stephen L. Lapointe, Randall P. Niedz, B. Wade French, Adriano E. Pereira, Deborah L. Finke, Kent S. Shelby, Thomas A. Coudron

**Affiliations:** 1 Division of Plant Sciences, University of Missouri, Columbia, Missouri 65211 USA; 2 Plant Genetics Research Unit, USDA-Agricultural Research Service, Columbia, Missouri 65211 USA; 3 U.S. Horticultural Research Laboratory, USDA-Agricultural Research Service, Fort Pierce, Florida 34945 USA; 4 North Central Agricultural Research Laboratory, USDA-Agricultural Research Service, Brookings, South Dakota 57006 USA; 5 Biological Control of Insects Research Laboratory, USDA-Agricultural Research Service, Columbia, Missouri 65203 USA

Correction to: *Scientific Reports* 10.1038/s41598-019-39709-x, published online 06 March 2019

The information in this Article is incomplete. The text for the Competing Interest statement,

‘The authors declare no competing interests.’

should read:

‘The authors declare no competing interests. Frontier Scientific Services (Newark, Delaware, USA) intends to market this diet.’

The proportions of all ingredients in the NCRMO-1 diet formulation are provided in Table 1.

**Table 1:** An artificial diet for NCR larvae (107 g).

Ingredients	WCRMO-1^1^	NCRMO-1
Egg powder	—	3.0 g
Glucose	—	1.0 g
Casein	2.5 g	3.0 g
Wheat germ (raw, ground)	5.5 g	6.0 g
Cellulose	1.5 g	1.0 g
Agar	1.5 g	1.5 g
Corn root powder	1.5 g	—
Sucrose	2.5 g	—
Linseed oil, raw	25 µl	—
Wheat germ oil	25 µl	—
Cholesterol	6 mg	6 mg
Wesson's salt mix	0.93 g	0.93 g
Vanderzant Vitamin mix	0.9 g	0.9 g
Methyl paraben	0.1 g	0.1 g
Sorbic acid	64 mg	64 mg
Potassium hydroxide (10%)	3.5 ml	3.5 ml
Streptomycin (12.8 mg/ml)	6.4 mg	6.4 mg
Chlortetracycline (10.0 mg/ml)	6.4 mg	6.4 mg
Distilled water	88 ml	88 ml
Green food coloring	64 µl	64 µl

Reference

1 Huynh, M. P. *et al*. Diet improvement for western corn rootworm (Coleoptera: Chrysomelidae) larvae. *PloS one* **12**, e0187997 (2017).

