# Supplementary material for: Multidimensional approach to formulating a specialized diet for northern corn rootworm larvae
Source: Sci Rep. 2019 Mar 6;9:3709. doi: 10.1038/s41598-019-39709-x (PMC6403226; doi:10.1038/s41598-019-39709-x)
Supplement: Supplementary file 1 — Supplementary Information [file 41598_2019_39709_MOESM1_ESM.docx]

**Supplementary Information**

**Multidimensional approach to formulating a specialized diet for northern corn rootworm larvae**

Man P. Huynh^1*^, Bruce E. Hibbard^2^, Stephen L. Lapointe^3^, Randall P. Niedz^3^, B. Wade French^4^, Adriano E. Pereira^2^, Deborah L. Finke^1^, Kent S. Shelby^5^, Thomas A. Coudron^5^

^1^Division of Plant Sciences, University of Missouri, Columbia, Missouri, 65211, USA

^2^Plant Genetics Research Unit, USDA-Agricultural Research Service, Columbia, Missouri, 65211, USA

^3^U. S. Horticultural Research Laboratory, USDA-Agricultural Research Service, Fort Pierce, Florida, 34945, USA

^4^North Central Agricultural Research Laboratory, USDA-Agricultural Research Service, Brookings, South Dakota, 57006, USA

^5^Biological Control of Insects Research Laboratory, USDA-Agricultural Research Service, Columbia, Missouri, 65203, USA

*Corresponding author: mphd32@missouri.edu

**Supplementary Methods:**

**Insect egg sterilization.** NCR eggs in 70 mesh sieved-soil were incubated at a constant 25^o^C in complete darkness and then washed out of the soils though a 60 mesh sieve (Hogentogler & Co. Inc., Columbia, MD) with water after the first eggs hatched. The remaining eggs were surface-treated using a procedure described by Pleau et al.[^1^](#_ENREF_1). Washed eggs were first held in undiluted lysol^®^ (Reckitt Benckiser, LLC, Parsippany, NJ) for 3 min and then triple rinsed with distilled water. Next, the eggs were held in 10% formalin (HT501128, Sigma Aldrich, St. Louis, MO) for 3 min and then triple rinsed with distilled water. Finally, the eggs were dispensed onto a coffee filter paper (Pure Brew, Rockline Industries, Sheboygan, WI) that was placed inside a 16 oz. cup (LG8RB-0090, Solo Cup Company, Lake Forest, IL) with a lid (DM16R-0090, Solo Cup Company) containing several holes made by a number zero insect pin. The eggs were then incubated at 25^o^C in darkness. Larvae that hatched within 24 hours were used for the insect bioassays.

**Diet preparation**. Diets were made using a procedure described in [Pleau et al.^1^](#_ENREF_1), with some modifications by [Huynh et al.^2^](#_ENREF_2). The solution of distilled water and agar (A7002, Sigma-Aldrich) in a 400 ml glass beaker was boiled in a microwave for 2 minutes and then poured into a blender (Hamilton Beach, Inc., Model 51101BZ) and placed in a biological safety cabinet (Nuaire, Biological safety cabinet). When the molten agar had cooled to 65^o^C, dry diet ingredients, i.e., corn gluten meal (49760, Sigma-Aldrich), cottonseed meal (Down-To-Earth, Eugene, OR), plant protein (Perfect supplements, Coventry, RI), whey protein (ON, Downers Grove, IL), perfect amino (Bodyhealth, Clearwater, FL), yeast extract (BP1422, Fisher Scientific, Fair Lawn, NJ), egg powder (Judee’s gluten free, Columbus, OH), casein (1100, Bio-Serv), wheat germ (1661, Bio-Serv, Flemington, NJ), cellulose (3425, Bio-Serv), glucose (D16, Fisher Scientific), sucrose (04821721, MP Biomedicals, Santa Ana, CA), corn root powder (USDA-ARS, Columbia, MO), salt mix (F8680, Bio-Serv), vitamin mix (V1007, Sigma-Aldrich), methyl paraben (H5501, Sigma-Aldrich), cholesterol (C8503, Sigma-Aldrich), and sorbic acid (S1626, Sigma-Aldrich)), were added (if applicable) to the blender and mixed thoroughly for 30 seconds. Next, liquid ingredients, i.e., linseed oil (430021, Sigma-Aldrich), wheat germ oil (W1000, Sigma-Aldrich), streptomycin (612240500, Across, Morris Plains, New Jersey), chlortetracycline (C4881, Sigma-Aldrich), green food coloring (Butler, Lancaster, PA) if applicable, were added to the mixture and blended for 30 seconds. Bio-Serv has sold their insect diets to Frontier Agricultural Sciences and all Bio-Serv ingredients can be found at <http://www.insectrearing.com/products/indiets.html>. The pH of the diet was monitored with indicator strips (Whatman^®^ 09-876-18, GE Healthcare Bio-Sciences, Pittsburg, PA) and adjusted a pH of 9.0 by the addition of 10% KOH (w/v) (P250, Fisher Scientific). The mixture was poured into a 750 ml glass beaker and slowly stirred at 65^o^C on a stirring hot plate (Thermo scientific, Cimarec^TM^). Using a repeater pipette (Eppendorf repeater plus), the diet mixture was pipetted into each well (200 µl/well) of a 96-well plate (3370, Corning Inc., Corning, NY) and then excess moisture was allowed to evaporate for 30 min. Diet plates were stored at 4^o^C and used within a week.

**Supplementary References**

1 Pleau, M. J., Huesing, J. E., Head, G. P. & Feir, D. J. Development of an artificial diet for the western corn rootworm. *Entomol. Exp. Appl.* **105**, 1-11 (2002).

2 Huynh, M. P. *et al.* Diet improvement for western corn rootworm (Coleoptera: Chrysomelidae) larvae. *PloS one* **12**, e0187997 (2017).

3 Cornell, J. A. *Experiments with mixtures: designs, models, and the analysis of mixture data, 3rd ed.* (John Wiley & Sons, 2002).

**Supplementary Table S1.** Diet blends of 8 components varied in the mixture exploratory design to rear northern corn rootworm larvae.

| Diet blend # | Agar  (g) | Sucrose  (g) | Wheat germ  (g) | Casein  (g) | Cellulose  (g) | Corn root  (g) | Wheat germ oil  (ml) | Linseed oil  (ml) |
| --- | --- | --- | --- | --- | --- | --- | --- | --- |
| 1 | 1 | 5 | 8 | 0 | 1.85 | 0 | 0.1 | 0.1 |
| 2 | 1 | 5 | 7.95 | 0 | 0 | 2 | 0 | 0.1 |
| 3 | 2 | 0 | 6.95 | 5 | 0 | 2 | 0 | 0.1 |
| 4 | 2 | 0 | 1.85 | 5 | 5 | 2 | 0.1 | 0.1 |
| 5 | 2 | 5 | 1.95 | 5 | 0 | 2 | 0.1 | 0 |
| 6 | 1 | 5 | 0 | 5 | 4.95 | 0 | 0 | 0.1 |
| 7 | 2 | 5 | 1.95 | 0 | 5 | 2 | 0 | 0.1 |
| 8 | 2 | 0.95 | 8 | 0 | 5 | 0 | 0.1 | 0 |
| 9 | 1 | 5 | 8 | 0.05 | 0 | 2 | 0 | 0 |
| 10 | 1.48 | 2.74 | 5.18 | 2.74 | 2.74 | 1.07 | 0.05 | 0.05 |
| 11 | 1.48 | 2.74 | 5.18 | 2.74 | 2.74 | 1.07 | 0.05 | 0.05 |
| 12 | 1 | 0 | 7.95 | 0 | 5 | 2 | 0.1 | 0 |
| 13 | 2 | 5 | 2.05 | 0 | 5 | 2 | 0 | 0 |
| 14 | 2 | 0 | 8 | 5 | 1.05 | 0 | 0 | 0 |
| 15 | 1 | 0 | 2.85 | 5 | 5 | 2 | 0.1 | 0.1 |
| 16 | 1.48 | 2.74 | 5.18 | 2.74 | 2.74 | 1.07 | 0.05 | 0.05 |
| 17 | 2 | 5 | 3.95 | 5 | 0 | 0 | 0.1 | 0 |
| 18 | 1 | 0 | 7.95 | 5 | 0 | 2 | 0.1 | 0 |
| 19 | 1 | 5 | 0.05 | 5 | 5 | 0 | 0 | 0 |
| 20 | 2 | 0 | 8 | 0.95 | 5 | 0 | 0 | 0.1 |
| 21 | 2 | 5 | 8 | 0.85 | 0 | 0 | 0.1 | 0.1 |
| 22 | 1.48 | 2.74 | 5.18 | 2.74 | 2.74 | 1.07 | 0.05 | 0.05 |
| 23 | 2 | 5 | 1.95 | 5 | 0 | 2 | 0.1 | 0 |
| 24 | 1 | 5 | 0.05 | 5 | 5 | 0 | 0 | 0 |

**Supplementary Table 2.** Diet blends of 8-proteins (in grams) varied in the mixture screening design to rear northern corn rootworm larvae.

| Diet blend # | Corn gluten meal | Cottonseed meal | Casein | Egg powder | Plant protein | Perfect Amino | Yeast extract | Whey protein |
| --- | --- | --- | --- | --- | --- | --- | --- | --- |
| 1 | 0.38 | 0.38 | 0.38 | 0.38 | 0.37 | 0.37 | 0.37 | 0.37 |
| 2 | 0.43 | 0 | 0.43 | 0.43 | 0.43 | 0.43 | 0.43 | 0.42 |
| 3 | 0 | 0.43 | 0.43 | 0.43 | 0.43 | 0.43 | 0.43 | 0.42 |
| 4 | 0.43 | 0.43 | 0.43 | 0.43 | 0 | 0.43 | 0.43 | 0.42 |
| 5 | 0.43 | 0.43 | 0.43 | 0 | 0.43 | 0.43 | 0.43 | 0.42 |
| 6 | 0.38 | 0.38 | 0.38 | 0.38 | 0.37 | 0.37 | 0.37 | 0.37 |
| 7 | 0 | 0 | 0 | 0 | 0 | 0 | 3 | 0 |
| 8 | 0.43 | 0.43 | 0.43 | 0.43 | 0.43 | 0 | 0.43 | 0.42 |
| 9 | 0.38 | 0.38 | 0.38 | 0.38 | 0.37 | 0.37 | 0.37 | 0.37 |
| 10 | 3 | 0 | 0 | 0 | 0 | 0 | 0 | 0 |
| 11 | 0.38 | 0.38 | 0.38 | 0.38 | 0.37 | 0.37 | 0.37 | 0.37 |
| 12 | 0.19 | 0.19 | 0.19 | 0.19 | 0.19 | 1.67 | 0.19 | 0.19 |
| 13 | 0 | 0 | 0 | 3 | 0 | 0 | 0 | 0 |
| 14 | 0.19 | 0.19 | 0.19 | 1.67 | 0.19 | 0.19 | 0.19 | 0.19 |
| 15 | 0.43 | 0.43 | 0 | 0.43 | 0.43 | 0.43 | 0.43 | 0.42 |
| 16 | 1.67 | 0.19 | 0.19 | 0.19 | 0.19 | 0.19 | 0.19 | 0.19 |
| 17 | 0 | 0 | 3 | 0 | 0 | 0 | 0 | 0 |
| 18 | 0.19 | 0.19 | 1.67 | 0.19 | 0.19 | 0.19 | 0.19 | 0.19 |
| 19 | 0 | 0 | 0 | 0 | 0 | 0 | 0 | 3 |
| 20 | 0 | 0 | 0 | 0 | 0 | 3 | 0 | 0 |
| 21 | 0.19 | 1.67 | 0.19 | 0.19 | 0.19 | 0.19 | 0.19 | 0.19 |
| 22 | 0.19 | 0.19 | 0.19 | 0.19 | 0.19 | 0.19 | 0.19 | 1.67 |
| 23 | 0.43 | 0.43 | 0.43 | 0.43 | 0.43 | 0.43 | 0 | 0.42 |
| 24 | 0 | 0 | 0 | 0 | 3 | 0 | 0 | 0 |
| 25 | 0.19 | 0.19 | 0.19 | 0.19 | 1.67 | 0.19 | 0.19 | 0.19 |
| 26 | 0.19 | 0.19 | 0.19 | 0.19 | 0.19 | 0.19 | 1.67 | 0.19 |
| 27 | 0.38 | 0.38 | 0.38 | 0.38 | 0.37 | 0.37 | 0.37 | 0.37 |
| 28 | 0 | 3 | 0 | 0 | 0 | 0 | 0 | 0 |
| 29 | 0.43 | 0.43 | 0.43 | 0.43 | 0.43 | 0.43 | 0.42 | 0 |
| 30 | 3 | 0 | 0 | 0 | 0 | 0 | 0 | 0 |

**Supplementary Table 3.** Components that were held constant in diets used to rear western corn rootworm larvae in the 8-protein mixture experiment.

| **Components** | **Amount** |
| --- | --- |
| 1. Agar | 1.5 g |
| 1. Cellulose | 1.0 g |
| 1. Chlortetracycline (10 mg/ml) | 6.4 mg |
| 1. Cholesterol | 6.0 mg |
| 1. Distilled water | 88 ml |
| 1. Food coloring | 6.4 mg |
| 1. Glucose | 1.0 g |
| 1. Methyl paraben | 0.1 g |
| 1. Potassium hydroxide (10%) | 3.5 ml |
| 1. Sorbic acid | 6.4 mg |
| 1. Streptomycin (12.8 mg/ml) | 6.4 mg |
| 1. Vanderzant vitamin mix | 0.90 g |
| 1. Wesson's salt mix | 0.93 g |
| 1. Wheat germ, ground | 6.0 g |

**Supplementary Table 4.** Mixture-amount design points to determine the optimum combination of key protein ingredients. The proportions of each mixture component (casein, egg powder, and whey) that make up the total amount of protein in the diet (casein (g) + egg powder (g) + whey (g)) are shown.

| Diet blend # | Proportion of mixture components | | | Total amount of mixture  [Casein + Egg powder + Whey] |
| --- | --- | --- | --- | --- |
|  | Casein | Egg powder | Whey |  |
| 1 | 0 | 0.5 | 0.5 | 6 |
| 2 | 0.16 | 0.16 | 0.67 | 2.25 |
| 3 | 0.16 | 0.67 | 0.16 | 2.25 |
| 4 | 0.5 | 0 | 0.5 | 6 |
| 5 | 1 | 0 | 0 | 3.5 |
| 6 | 0.5 | 0.5 | 0 | 6 |
| 7 | 1 | 0 | 0 | 6 |
| 8 | 0 | 1 | 0 | 6 |
| 9 | 1 | 0 | 0 | 1 |
| 10 | 0.5 | 0 | 0.5 | 1 |
| 11 | 0.67 | 0.16 | 0.16 | 4.75 |
| 12 | 0.16 | 0.16 | 0.67 | 4.75 |
| 13 | 0.5 | 0.5 | 0 | 1 |
| 14 | 0 | 1 | 0 | 6 |
| 15 | 0 | 1 | 0 | 3.5 |
| 16 | 0 | 0.5 | 0.5 | 1 |
| 17 | 1 | 0 | 0 | 1 |
| 18 | 0 | 0.5 | 0.5 | 3.5 |
| 19 | 0.5 | 0.5 | 0 | 3.5 |
| 20 | 0.5 | 0.5 | 0 | 1 |
| 21 | 0.5 | 0 | 0.5 | 3.5 |
| 22 | 0 | 0 | 1 | 1 |
| 23 | 0 | 0.5 | 0.5 | 1 |
| 24 | 0 | 0 | 1 | 1 |
| 25 | 0.16 | 0.67 | 0.16 | 4.75 |
| 26 | 0 | 1 | 0 | 1 |
| 27 | 0 | 0 | 1 | 3.5 |
| 28 | 0 | 0 | 1 | 6 |
| 29 | 1 | 0 | 0 | 6 |
| 30 | 0 | 1 | 0 | 1 |
| 31 | 0.67 | 0.16 | 0.16 | 2.25 |
| 32 | 0 | 0 | 1 | 6 |

**Supplementary Table 5.** *p*-values, regression coefficients and response surface model fitting diagnostic statistics for NCR larval responses to 8-component diet mixtures. A: agar, B: sucrose, C: wheat germ, D: casein, E: cellulose, F: corn root powder, G: wheat germ oil, H: linseed oil. ^a^Expressed in coded forms. Mixture component coding is U_Pseudo[^3^](#_ENREF_3).

|  | Weight  *p*-values | Regression coefficients^a^ | % Molt  *p*-values | Regression coefficients | % Survival  *p*-values | Regression coefficients |
| --- | --- | --- | --- | --- | --- | --- |
| Model | <0.0001 | - | <0.0001 | - | <0.0001 | - |
| Linear mixture | <0.0001 | - | <0.0001 | - | <0.0001 | - |
| A | - | -2.29 | - | -9.28 | - | -0.62 |
| B | - | 2.13 | - | 1.53 | - | 1.12 |
| C | - | -0.89 | - | -0.53 | - | 1.82 |
| D | - | 0.22 | - | 9.41 | - | 1.04 |
| E | - | -0.27 | - | 1.56 | - | 1.04 |
| F | - | -1.31 | - | -3.41 | - | 0.30 |
| G | - | -21.47 | - | -29.7 | - | -4.25 |
| H | - | -12.3 | - | -58.09 | - | 1.06 |
| B^2^ | 0.0354 | -5.97 | - | - | - | - |
| C^2^ | - |  | - | - | <0.0006 | -1.92 |
| D^2^ | 0.0485 | -5.73 | <0.0001 | -32.43 | - | - |
|  |  |  |  |  |  |  |
| Lack of fit | 0.4138 |  | <0.0001 |  | 0.2778 |  |
| Model type | Quadratic (reduced) |  | Quadratic (reduced) |  | Quadratic (reduced) |  |
| Transformation | Base 10 log |  | Base 10 log |  | N/A |  |
|  |  |  |  |  |  |  |
| R^2^ | 0.9633 |  | 0.9644 |  | 0.9159 |  |
| R^2^_adj_ | 0.9378 |  | 0.9440 |  | 0.8679 |  |
| R^2^_pred_ | 0.8296 |  | 0.8955 |  | 0.7550 |  |

**Supplementary Table 6.** *p*-values, regression coefficients and response surface model fitting diagnostic statistics for WCR larval responses to 8-protein diet mixtures. A: corn gluten meal, B: cottonseed meal, C: casein, D: plant protein, E: whey protein, F: perfect amino, G: yeast extract, H: egg powder. ^a^Expressed in coded forms. Mixture component coding is L_Pseudo[^3^](#_ENREF_3).

|  | Weight  *p*-values | Regression coefficients^a^ | % Molt  *p*-values | Regression coefficients | % Survival  *p*-values | Regression coefficients |
| --- | --- | --- | --- | --- | --- | --- |
| Model | <0.0001 | - | <0.0001 | - | <0.0001 | - |
| Linear mixture | <0.0001 | - | <0.0001 | - | <0.0001 | - |
| A | - | -1.69 | - | -0.02 | - | 0.24 |
| B | - | -1.40 | - | 0.5 | - | 0.90 |
| C | - | -0.79 | - | 0.91 | - | 0.96 |
| D | - | -1.49 | - | 1.78 | - | 1.86 |
| E | - | -0.34 | - | 1.78 | - | 1.00 |
| F | - | 0.12 | - | -0.11 | - | 0.20 |
| G | - | -1.40 | - | 0.58 | - | 0.72 |
| H | - | 0.88 | - | 0.94 | - | 0.99 |
| A*D | 0.0029 | 5.95 | - | - | - | - |
| A*F | <0.0001 | -17.27 | - | - | - | - |
| A*H | 0.0044 | 5.63 | - | - | - | - |
| D^2^ | - | - | 0.0084 | -1.46 | <0.0001 | -1.15 |
| E^2^ | 0.0198 | -0.73 | 0.0612 | -0.99 | - | - |
| F^2^ | 0.0021 | -1.61 | - | - | - | - |
|  |  |  |  |  |  |  |
| Lack of fit | 0.0649 |  | 0.0067 |  | 0.0393 |  |
| Model type | Quadratic (reduced) |  | Quadratic (reduced) |  | Quadratic (reduced) |  |
| Transformation | N/A |  | Square root |  | N/A |  |
|  |  |  |  |  |  |  |
| R^2^ | 0.9617 |  | 0.8805 |  | 0.9277 |  |
| R^2^_adj_ | 0.9346 |  | 0.8267 |  | 0.9002 |  |
| R^2^_pred_ | 0.7343 |  | 0.6317 |  | 0.6040 |  |

**Supplementary Table 7.** *p*-values, regression coefficients and response surface model fitting diagnostic statistics for WCR larval responses to 3-protein mixture-amount experiment**.** A: egg powder, B: whey protein, C: casein. ^a^Expressed in coded forms, ^b^Concentration. Mixture component coding is L_Pseudo[^3^](#_ENREF_3).

|  | Weight  *p*-values | Regression coefficients^a^ | % Molt  *p*-values | Regression coefficients | % Survival  *p*-values | Regression coefficients |
| --- | --- | --- | --- | --- | --- | --- |
| Model | <0.0001 | - | <0.0001 | - | <0.0001 | - |
| Linear mixture | <0.0122 | - | <0.0207 | - | <0.0001 | - |
| A | - | 0.21 | - | 0.79 | - | 0.96 |
| B | - | 0.13 | - | 0.71 | - | 0.98 |
| C | - | 0.14 | - | 0.68 | - | 0.87 |
| A*B | - | - | 0.0262 | 0.35 | - | - |
| A*C | 0.0028 | 0.39 | 0.0001 | 0.75 | 0.0387 | -0.12 |
| B*C | - | - | 0.0203 | 0.36 | 0.0318 | 0.11 |
| A*Conc.^b^ | <0.0001 | 0.12 | 0.0004 | 0.14 | - | - |
| B* Conc. | 0.0934 | -0.04 | - | - | - | - |
| C* Conc. | - | - | <0.0001 | 0.21 | 0.0030 | 0.03 |
| C* Conc.^2^ | - | - | - | - | 0.0093 | 0.05 |
| A*C* Conc. | - | - | 0.0025 | -0.67 | - | - |
|  |  |  |  |  |  |  |
|  |  |  |  |  |  |  |
| Lack of fit | 0.0368 |  | 0.1075 |  | 0.6601 |  |
| Model type | Quadratic mixture (reduced) x linear amount |  | Quadratic mixture (reduced) x linear amount |  | Quadratic mixture (reduced) x quadratic amount (reduced) |  |
| Transformation | N/A |  | N/A |  | N/A |  |
|  |  |  |  |  |  |  |
| R^2^ | 0.6714 |  | 0.8369 |  | 0.6964 |  |
| R^2^_adj_ | 0.6057 |  | 0.7776 |  | 0.6173 |  |
| R^2^_pred_ | 0.4407 |  | 0.6490 |  | 0.4138 |  |

**
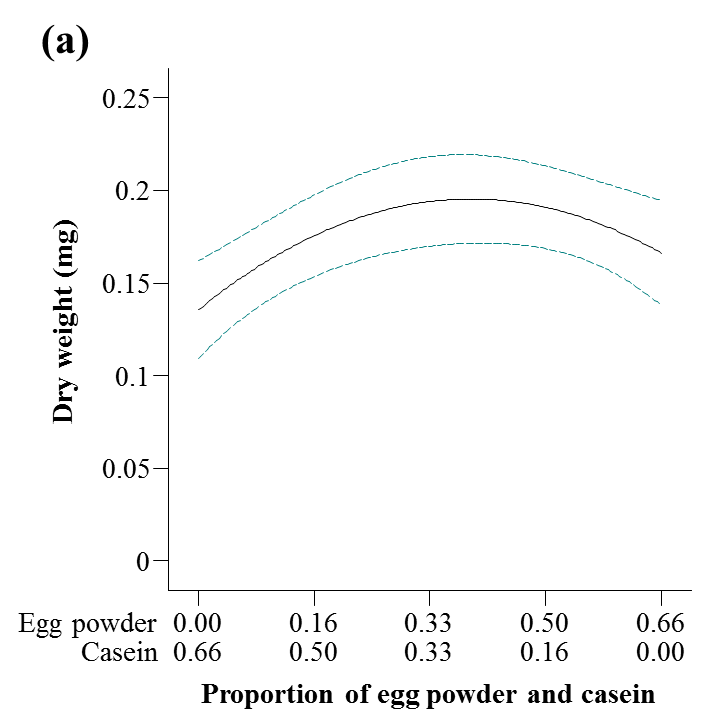

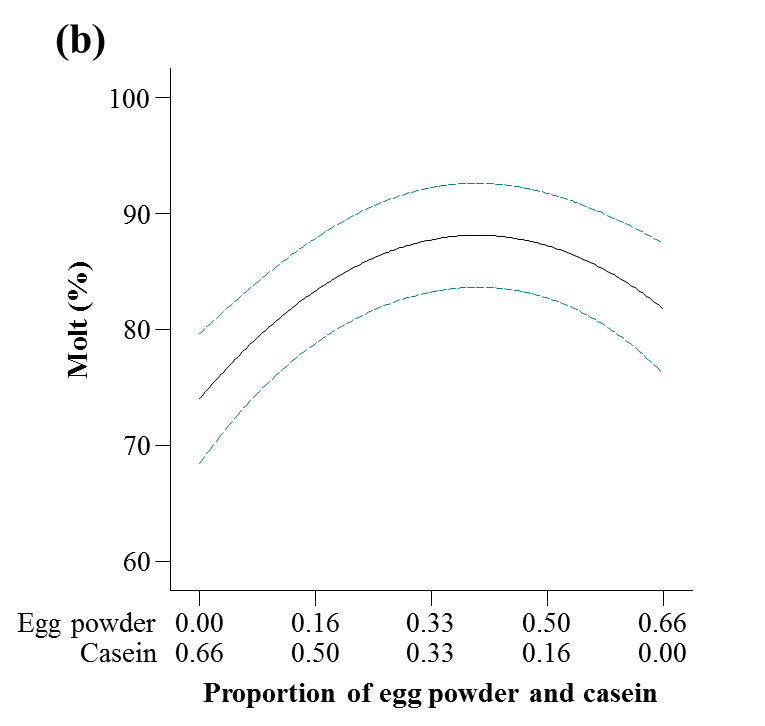

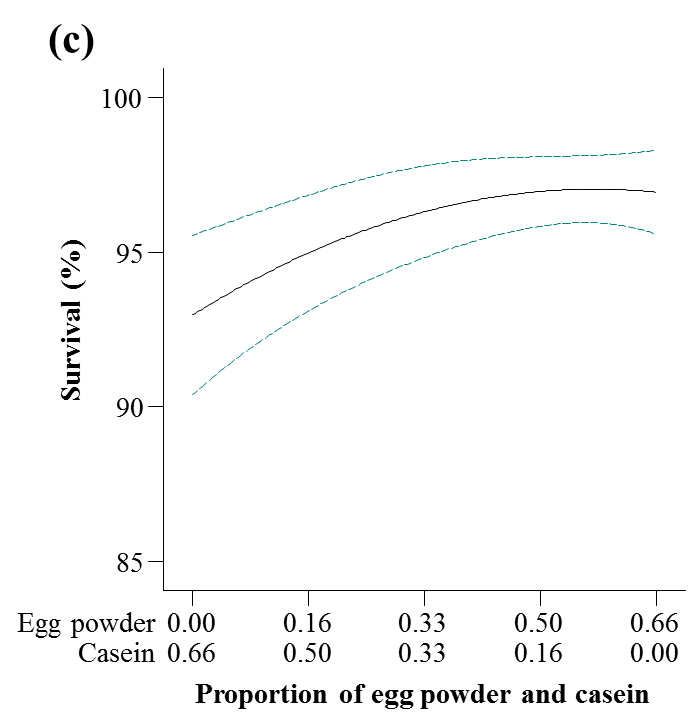
**

**Supplementary Figure 1.** Nonlinear blending effects of casein × egg powder from the mixture-amount experiment. (a) weight, (b) molting, and (c) survival. Dotted lines indicate 95% confidence interval bands. Proportion of whey protein = 0.34. Total amount of mixture = 3 grams.
